# The Antibiofilm Nanosystems for Improved Infection Inhibition of Microbes in Skin

**DOI:** 10.3390/molecules26216392

**Published:** 2021-10-22

**Authors:** Yin-Ku Lin, Shih-Chun Yang, Ching-Yun Hsu, Jui-Tai Sung, Jia-You Fang

**Affiliations:** 1 Department of Traditional Chinese Medicine, Chang Gung Memorial Hospital, Keelung 204, Taiwan; lin1266@cgmh.org.tw; 2 School of Traditional Chinese Medicine, Chang Gung University, Kweishan, Taoyuan 333, Taiwan; 3 Pharmaceutics Laboratory, Graduate Institute of Natural Products, Chang Gung University, Kweishan, Taoyuan 333, Taiwan; phageyang@gmail.com (S.-C.Y.); d000011794@cgu.edu.tw (J.-T.S.); 4 Department of Nutrition and Health Sciences, Chang Gung University of Science and Technology, Kweishan, Taoyuan 333, Taiwan; cyhsu@mail.cgust.edu.tw; 5 Research Center for Food and Cosmetic Safety and Research Center for Chinese Herbal Medicine, Chang Gung University of Science and Technology, Kweishan, Taoyuan 333, Taiwan; 6 Department of Anesthesiology, Chang Gung Memorial Hospital, Kweishan, Taoyuan 333, Taiwan

**Keywords:** biofilm, microbe, nanoparticle, skin, infection, resistance

## Abstract

Biofilm formation is an important virulence factor for the opportunistic microorganisms that elicit skin infections. The recalcitrant feature of biofilms and their antibiotic tolerance impose a great challenge on the use of conventional therapies. Most antibacterial agents have difficulty penetrating the matrix produced by a biofilm. One novel approach to address these concerns is to prevent or inhibit the formation of biofilms using nanoparticles. The advantages of using nanosystems for antibiofilm applications include high drug loading efficiency, sustained or prolonged drug release, increased drug stability, improved bioavailability, close contact with bacteria, and enhanced accumulation or targeting to biomasses. Topically applied nanoparticles can act as a strategy for enhancing antibiotic delivery into the skin. Various types of nanoparticles, including metal oxide nanoparticles, polymeric nanoparticles, liposomes, and lipid-based nanoparticles, have been employed for topical delivery to treat biofilm infections on the skin. Moreover, nanoparticles can be designed to combine with external stimuli to produce magnetic, photothermal, or photodynamic effects to ablate the biofilm matrix. This study focuses on advanced antibiofilm approaches based on nanomedicine for treating skin infections. We provide in-depth descriptions on how the nanoparticles could effectively eliminate biofilms and any pathogens inside them. We then describe cases of using nanoparticles for antibiofilm treatment of the skin. Most of the studies included in this review were supported by in vivo animal infection models. This article offers an overview of the benefits of nanosystems for treating biofilms grown on the skin.

## 1. Introduction

The skin is the largest organ in the human body, and it serves as a barrier protecting the body from the invasion of foreign organisms and toxic substances. As an interface with the outer environment, the barrier function of intact skin prevents the ingress of foreign microbes but offers a home for commensal microbiota. The skin is colonized by a diverse microbiome that includes bacteria, fungi, and viruses [1]. The influence of skin microbiota on the management of cutaneous health and disease has been widely investigated in recent years [2]. The distribution and growth of the skin microbiome depend on the microenvironment of the skin region. There are differences in the microbial population, diversity, and evenness among sites that are moist, dry, and sebaceous. In addition, the cruel landscape of the skin, especially its nutrient-poor and acidic microenvironment, can lead to a specific distribution of specified microorganisms on the skin. Pathogenic microbes can produce infectious diseases that threaten human life. Infections induced by microorganisms are the second-highest cause of global human death [3]. The emergence of microbial resistance to antibiotics is becoming a major problem due to their broad use and abuse [4]. Most infections are induced by bacteria. Bacteria that cause infections can exist in different forms—planktonic, intracellular, and biofilm states—in the human body. Planktonic bacteria are free-living microbes that exist as floating microorganisms in their respective environments. The move of bacteria into host cells is a stage for eliciting an intracellular infection. This issue mainly occurs in immune cells such as macrophages and neutrophils. Bacteria can facilely survive in the host cells for a prolonged time. Antibiotics usually exhibit limited activity to kill intracellular microbes. A high antibiotic concentration is needed to eliminate intracellular bacteria, resulting in possible adverse effects and toxicity [5].

Besides host cells, a biofilm can serve as a shelter for pathogenic microorganisms to hinder the attack from antibiotics and the host clearance system. A biofilm is an aggregate of microbes in which the cells are embedded in a self-produced matrix of extracellular polymeric substances (EPS) consisting of proteins, polysaccharides, lipids, and deoxyribonucleic acids (DNA) [6]. The sessile community of microbes in a biofilm accounts for the fact that the cells experience cell-to-cell contact, called quorum sensing (QS). This system is a major component of bacterial communication and acts as a language for the interactions among the neighboring bacteria that respond to the extracellular, diffusible small molecule signals. The production of the signals helps the microorganisms with overwhelming the host’s defense by releasing exotoxin [7]. The microenvironment of EPS is deficient in oxygen, contributing to anaerobic glycolysis, hypoxia, and ion channel turbulence [8]. A biofilm is even more acidic than healthy tissue. A biofilm’s resistance to antibiotics is caused by a dormant phenotype induced by adaptation to an anoxic microenvironment and nutrient deprivation. The metabolic level of bacteria is low and cell division occurs at a down-regulated rate, producing slow-growing microbes that can tolerate antimicrobial treatment [9]. The rigid structure of biofilms formed by EPS establishes a barrier to retard the penetration of antimicrobial agents. Many conventional antibiotics fail to treat infections due to the formation of biofilms by pathogenic microorganisms [10]. Biofilms are the archcriminal means of many chronic and obstinate infection disorders. It is estimated that biofilms are involved in approximately 80% of bacterial infections and cause more than 500,000 deaths per year globally [11]. Biofilms indicate the threat of increased bacterial virulence for infection. There has been no biofilm-specific therapy until now. The development of new therapeutic strategies against biofilms, especially for drug-resistant bacteria, is critically necessary.

Most antimicrobial agents aim to treat planktonic pathogens. These agents are usually ineffective at treating biofilm infections [12]. The current treatment for biofilm infections is the use of combined antibiotics [13]. However, the toxicity and increased bacterial resistance accompanied by this combined use have raised the search for other biofilm infection treatments. Recently, the application of nanomedicine has started an innovative era in antibiofilm therapy. In a broad definition, nanoparticles are a class of materials regarded as particles sized 1 to 100 nm (usually containing particles sized several hundred nm), which have properties that are distinctively different from their bulk and molecular counterparts. Nanoparticles can be defined as having size-related biological properties that are significantly different from either fine particles or bulk materials. Nanoparticles have been successfully used for the delivery of antibiotics to prevent or treat bacterial colonization in biofilms. In addition to antibiotics, non-drug agents such as enzymes and essential oils can be loaded into nanocarriers for antibiofilm delivery [14]. Some nanoparticles themselves can also exhibit antibiofilm effects due to the inclusion of antibacterial materials such as metal oxides and cationic surfactants [15]. Their large surface area to mass, high reactivity, and surface functionalization have led to unique features for the efficient eradication of biofilm microorganisms [16]. These characteristics have been employed to design nanocarriers with controlled drug delivery, reduced drug toxicity, enzymatic attack prevention, improved drug stability, and facile EPS penetration. Due to their advantages for biofilm elimination, nanoparticles are an attractive approach to overcome the antibiotic resistance caused by biofilms.

Nanoparticles have been extensively employed for enhancing topical drug absorption because of the capability to improve epithelium permeability and bioavailability, thereby prolonging the bioactivity of drugs in the cutaneous nidus [17]. Nanosystems can promote skin delivery based on the affinity to the stratum corneum, facile formation of a drug reservoir, specific nanoparticle-skin interactions, and flexible shapes to squeeze into the skin [18]. Nanoparticles can be applied for wound healing and associated microbe infections due to the ability to prolong drug release and the broad distribution in the wound area [19]. Antimicrobial nanoparticles are suitable for application via topical routes to treat skin infections. This approach can increase the therapeutic efficacy and reduce the risk of systemic side effects. With the evolution of nanomedicine, the application of nanocarriers is expected to change the landscape of antibiofilm therapy. In this review, we highlighted recent advances in the antimicrobial activity of topically applied nanoparticles to treat biofilms. We mainly focused on studies on nanoparticle development during the past ten years for the eradication of biofilms on skin. The promising perspective in this emerging investigation was also discussed.

## 2. Cutaneous Microbiome

The skin is basically composed of three layers: the stratum corneum, the epidermis, and the dermis. The stratum corneum is the outermost layer of skin and contains terminally differentiated keratinocytes called squames, which are crosslinked to render the major barrier function of the skin. The epidermis and the dermis are viable layers that predominantly contain keratinocytes and fibroblasts, respectively. The skin’s surface is dry and acidic, with a pH of around 4.1 to 5.8 [20]. The top layer of the epidermis continuously releases 600,000−1,000,000 keratinized cells per hour for renewal of the skin every month [21]. It has been reported that about 10% of exfoliated cells are composed of bacteria that affect the skin microbiota’s composition [22]. The host microorganisms are also largely colonized in the folds and navel of the skin’s surface due to moist environments. The numbers of microbes in regions with deep folds, such as the groin and armpits, can be 10^6^ colony-forming units (CFU)/cm^2^, which is greater than the average number from the whole skin area (10^3^−10^4^ CFU/cm^2^), as determined by conventional culture methods [23]. Besides the exfoliated cells on the cutaneous surface, appendages such as hair follicles, sebaceous glands, and sweat glands are sites for the residence of microbiomes. These invaginations are associated with their own microbiota. Hair follicles are a potential site for microorganisms to deposit because of the grooved cuticle surface of the hair shaft. Compared to the skin, hair follicles favor microbial growth, as they are moist, well-perfused, and relatively UV-protected [24]. About 25% of cutaneous bacteria deposit in the hair follicles [25]. The bacterial aggregates in the hairs consist of the fungal strain *Malassezia* and bacterial strains such as *Propiobacterium*, *Cutibacterium*, and *Staphylococcus*, which are among the most abundant microbes in the scalp. These microorganisms violate hair follicles to induce furuncles, folliculitis, and hidradenitis suppurativa. Sebaceous glands are dominated by the *Propiobacterium* species. Other species, including *Staphylococcus* and *Corynebacterium*, are also largely colonized in sebaceous sites that are abundant with moisture. For antimicrobial treatments in the appendages, nanoparticles offer an efficient approach to specifically target these invaginations. Nanoparticles can aggregate in follicular openings and penetrate along the follicular duct when administered onto the skin’s surface. Some nanoparticles possess malleable properties to extrude into the appendages [26]. Lipophilic materials such as sebum have affinity for lipid-based nanoparticles.

**Figure 1:**
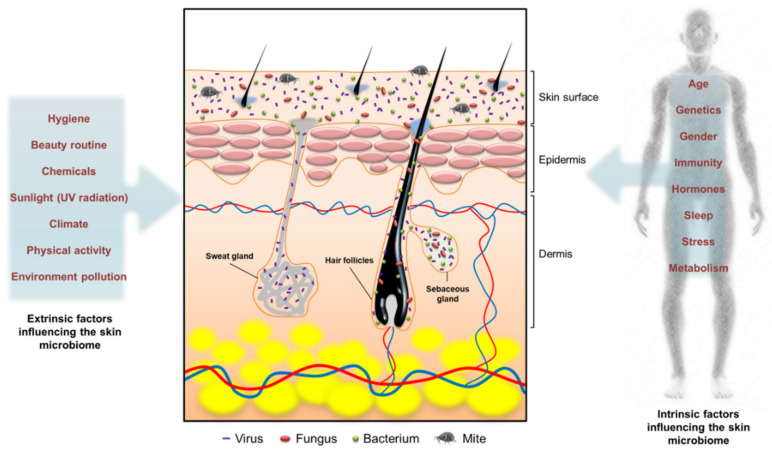
The intrinsic and extrinsic factors influencing the microbiome on the skin.

Both intrinsic and extrinsic factors can influence the type, colonization site, and strain of microbiota in skin tissue (Figure 1). Age, sex, host genotype, and immune reactivity are the intrinsic factors influencing the composition of skin microbe communities [27]. For instance, infant skin is known to have a poor barrier function and is prone to candidiasis infections [2]. Pathogenic infection by bacterial communities also contributes to infant skin homeostasis by affecting the inflammatory response. Extrinsic factors, including climate, occupation, the use of antibiotics, and hygiene, can modulate the colonization of skin microbiota. Soaps, cosmetics, and hygienic products are daily care products that affect the diversity of skin microbiota. These products can alter the condition of the skin barrier to modulate the microbiome. The diversity of skin microbial communities varies in inter- and intra-subjects. Generally, coagulase-negative staphylococci, especially *Staphylococcus epidermidis*, anaerobic *Cutibacterium acnes*, *Corynebacterium*, *Streptococcus*, *Acinetobacter*, and *Micrococcus*, are the main species on the skin. The representatives of *Staphylococcus*, *Cutibacterium*, and *Corynebacterium* contribute 45−80% of the whole cutaneous microbiota [21]. The microbiomes in skin biofilms are complex, having high microbe densities of 10^8^−10^11^ cells/g and many species [28].

*S. epidermidis* is regarded as the primary microorganism colonizer of the skin. This species is a skin commensal but can also be an opportunistic pathogen in barrier-disintegrated or immunocompromised skin [29]. The pathogenic nature of *S. epidermidis* depends upon the disease condition and the microbiome. In patients with atopic dermatitis (AD), a correlation has been found between symptom deterioration and the *S. epidermidis* quantity [30]. Virulent strains of *S. epidermidis* can produce biofilms to protect them from the host’s immune system, thereby increasing the resistance level. *S. aureus* is one of the main biofilm-forming pathogens found on the skin. This strain is an opportunistic microbe that can generate abscesses, cellulitis, folliculitis, and lymphangitis [31]. The drug-resistant S. aureus has a high probability of establishing a biofilm for developing into persistent and recurrent infections [9]. Multidrug-resistant *S. aureus*, especially methicillin-resistant *S. aureus* (MRSA), is a frequent cause of recurrent skin and soft tissue infections. MRSA represents 26% of all infections induced by *S. aureus* [32]. *C. acnes* is a gram-positive anaerobic bacterium that forms a biofilm which is more resistant to antibiotics than planktonic cells. *C. acnes* is reported to represent >30% of the facial microbes in acne patients [33]. *C. acnes* is also linked to a number of other infections, such as folliculitis, endocarditis, periodontitis, sarcoidosis, and medical catheter-related infections [34].

Non-bacterial microbes can also be isolated from the skin as commensal or opportunistic pathogens. Fungi and viruses are the non-bacterial microbes most commonly isolated from the skin. The dominating genus of fungi on the skin is *Malassezia*, which acts as a resident microbe in healthy skin. This fungus is especially prevalent in sebaceous glands [35]. Dandruff and seborrheic dermatitis are skin disorders that are mainly caused by the opportunistic *Malassezia* [36]. The *Malassezia* species is principally colonized in the core of the body and the arms. The fungal diversity is high on the foot, with *Aspergillus*, *Cryptococcus*, *Rhodotorula*, and *Epicoccum* all being found in this area. The profile of viruses residing in human skin is limited. Viruses classified as belonging to the Papillomaviridae, Circoviridae, and Polyomaviridae families have been identified on the skin of some populations, with high levels of variability [21].

## 3. Cutaneous Microbiome-Associated Skin Diseases

The skin serves as a formidable barrier to prevent the invasion of pathogens. When a barrier dysfunction occurs, microorganisms penetrate the skin to cause an imbalance between commensal and pathogens. This process is called dysbiosis [37], which is often driven by commensal species. Commensal microbes facilely locate in the lesions of atopic dermatitis (AD), acne, and chronic wounds to reveal an essential capacity on infection-induced biofilm and skin disease evolution.

### 3.1. Atopic Dermatitis (AD)

AD is a chronic autoimmune inflammatory skin disease that affects 10−30% of children and 1−3% of adults in the world [38]. AD causes inflamed and itchy skin with a predilection for skin flexure. It is characterized by intense pruritus, erythematous papules with excoriation, vesicles over erythematous skin, thickened plaques of skin, accentuated skin markings (lichenification), and fibrotic papules (prurigo nodularis). The symptoms of AD can cause barrier function defects, followed by the invasion of bacteria and allergens [39]. More than 90% of AD patients are largely colonized with *S. aureus* on both lesional and non-lesional sites [40]. An AD flare manifests a decline of microbial diversity and a dramatic increase in the relative abundance of *S. aureus*. The relative number of *S. aureus* correlates well with the severity of AD symptoms [41]. In addition to *S. aureus*, *S. epidermidis* appears in AD flares and predominates in less severe lesions. This suggests that AD can arise from dysbiosis without the invasion of pathogens prevailing in the community. A recent investigation unveiled that fungi play a fundamental role in protecting the skin from AD [41]. *Malassezia* peaks in prevalence in the oily skin of infants and adolescents; however, AD is less common in adults. The inhibition of skin surface lipids restricts the ecological competitiveness of *Malassezia*. Chng et al. [42] found a relative decrease in *Malassezia* population in AD with the rise of *S. aureus* colonization.

### 3.2. Acne

Acne vulgaris is the eighth-most prevalent disorder in the world and affects about 10% of the population, especially adolescents [43]. The *C. acnes*-associated biofilm in hair follicles elicits the symptoms of increased seborrhea, hyperkeratinization, erythema, and comedones in acne patients. Available drug therapies for treating acne vulgaris are limited, and none of them are regarded as a definite cure because of their side effects [44]. New perspectives in the treatment of acne focus on biofilm targeting and antibiotic resistance prevention. The pathogenesis of acne involves the dysbiosis of skin microbiota. The superiority of *C. acnes* over other microorganisms in the development of acne has led to the formation of *C. acnes* biofilms attached to the hair shaft and follicular epithelium, which raises the morbidity of acne patients [45]. Besides the interspecies dysbiosis of skin microbiota, the shift of intraspecies of *C. acnes* also affects acne pathogenesis. The most abundant ribotypes of *C. acnes* include RT1, RT2, and RT3, which display similar numbers in acne and healthy sebaceous units, whereas RT4 and RT5 are enriched in up to 40% of acne patients as compared to normal subjects [46].

### 3.3. Skin Wounds

Skin wounds represent a serious and overlooked global health problem. Untreated skin wounds are associated with the complications of long-term morbidity, amputation, and infection. Wound infection is the major obstacle to wound healing, resulting in the increased morbidity and mortality of patients. *S. aureus* is the most common pathogen found in wounds [47], and the presence of MRSA worsens the risk of wound management failure. Pathogenic microorganisms colonize the skin and hinder the healing of chronic wounds in elderly, obese, and diabetic patients. It has been found that more than 50% of diabetic foot ulcers are infected [1]. Wound healing is complicated by the infections caused by pathogens that are resistant to biocides and possess the ability to establish a biofilm [48]. Biofilms develop within the wound bed of chronic wounds, representing a physical barrier to wound closure. MRSA, *Pseudomonas aeruginosa*, and enterobacteria species can form chronic biofilms that are difficult to treat [49]. Biofilms in chronic wounds are usually multispecies, and it has been demonstrated that 51% of infected wounds have more than two microbes [50]. Burn wounds also give facile access to pathogenic microbes, enabling them to induce systemic infection. The presence of a biofilm in the burn wound site can delay healing. The specified microbial species of *P. aeruginosa*, *Streptococcus pyogenes*, and *Enterococcus* genus are readily identified in chronic wound biofilms [51]. The number of burn wound patients with MRSA biofilm infections is also continually increasing.

## 4. Biofilms

### 4.1. The Formation and Dispersion of Biofilms

**Figure 2:**
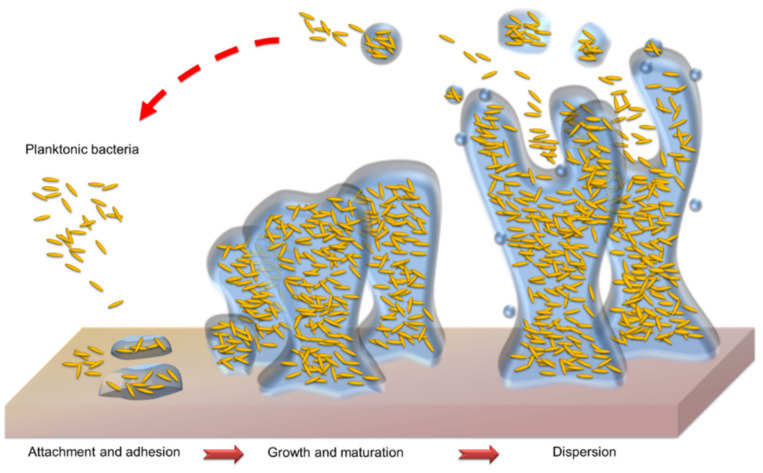
The process of the microbial biofilm establishment.

A biofilm is the 3D community of microbes adhering onto a surface and encased in a protective EPS. The lifecycle of a biofilm can be divided into four stages: the initial attachment of microbes to a surface, the formation of colonies on the surface, the maturation of colonies in the established biofilm, and microbial dispersal from the biofilm (Figure 2). At the first stage, planktonic bacteria migrate and adhere to surfaces such as skin. The adherent bacteria initiate biofilm production and become encased in a small quantity of EPS material. This attachment to the surface is mediated by adhesive surface proteins, fimbriae, and specific polysaccharides [52]. Bacterial adhesion prefers rougher and more lipophilic surfaces. At the second stage, the adherent bacteria exude EPS to irreversibly attach to the surface. Thus, the cell aggregate and matrix are established. The biofilm then becomes mature through the growth of microcolonies and the aqueous channel architecture. The complete biofilm reaches the maximum bacterial density with a 3D structure. Finally, the mature biofilm releases microcolonies from the main community. These microcolonies are capable of adhering to new surfaces to spread the infection. Fungal infections also contribute to the creation of biofilms to exacerbate infections. *Candida albicans* adheres onto the skin and the surfaces of medical catheters to form biofilms [53]. The hypha, a long filamentous structure, is a prominent characteristic of *C. albicans* biofilms. The hyphal filament penetrates the tissue to assist fungal biofilm establishment in superficial fungal infections [54].

### 4.2. The Components and Structure of Biofilms

The matrix of a biofilm predominantly comprises EPS and the cells. The EPS organization in the biomass is based on the intermolecular interaction between EPS components to govern the mechanical and physiological properties. EPS mediates the biomass architecture formation through a dynamic and continuous process, creating a spatial organization in which the microorganisms assemble in a microcolony [55]. The main constituent of the matrix is water, which contains the structural components of soluble proteins, polysaccharides, and extracellular DNA. Insoluble components including amyloids, cellulose, pili, and flagella are also included in the biomass [56]. Extracellular DNA is an important component in EPS for supporting the filamentous network stability of the biofilm. Channels and pores exist inside the biomass to form voids. These voids facilitate liquid transport. The matrix can capture the resources of nutrients that are present in the aqueous phase for biofilm growth. Nutrient capture is an essential process for microorganisms. This process relies on the passive sorption of sponge-like EPS, which affects the exchange of nutrients, gases, and other materials between the environment and the biofilm [57]. The substances that are sequestered from the aqueous phase are kept in the biofilm and regarded as sorbed.

The increased resistance to antimicrobial drugs or agents compared to planktonic cells is the emergent property of a biofilm. The biomass can be considered a fortress in which the antimicrobial tolerance and survival of desiccation form buttresses [58]. The EPS in a biofilm can quench the activity of antimicrobial agents that transfer into the biofilm in a form of suppression known as diffusion-reaction inhibition. This inhibition can involve chelation by complex formation, enzymatic biodegradation, oxidizing disinfectants, and the sacrificial responses of EPS. Dormancy and slow growth rates are bacterial survival approaches in biofilms exposed to antibiotics [59]. A substantial number of microbes in the stationary phase are included in a biofilm. These cells show a reduced susceptibility to many antibiotics that depend on the metabolism of bacteria for their activity. At least 1% of the bacteria in biofilms belong to the stationary phase and can tolerate antibiotics [60].

### 4.3. Treatment Strategies for Biofilm Eradication

Due to biofilms’ rigid and formidable architectures, most antibiotics cannot easily penetrate the biofilm. The biomass provides an environment for gene transfer between individual cells, thereby spreading the resistance to virulence [61]. Biofilm pathogens have 10 to 1000-fold greater resistance to antibiotics than the free-living pathogens [62]. The biofilm development inhibition and interference remain tenuous until now. Currently, some strategies have been utilized to conquer the antibiotic resistance of biofilms. These include mechanical, physical, and chemical methods. In order to resolve biofilm infections, water jets, photodynamic therapy (PDT), photothermal therapy (PTT), enzymes, antimicrobial peptides, cationic surfactants, and nanoparticles have been proved to be beneficial for the clearance of biofilms and the microbes inside them [63]. Procedures for the physical removal of biofilms by the local delivery of mechanic forces have been developed for a long time. The mechanical disruption of biomasses by water-based sprays and jets has been used for biofilm removal and irrigation, thereby evoking debridement of the infection and withdrawing the biofilm and necrotic tissue [64]. Water jets can incorporate antimicrobial agents for delivery to the biofilm, leading to a synergistic effect.

PDT mediates the killing of living cells with the combination of a light source and a photosensitizer. The photosensitizer creates reactive oxygen species (ROS) via excitement under a specified irradiation wavelength, which ultimately are toxic for bacteria and fungi [65]. Photosensitizers can be porphyrin derivatives, phthalocyanine, spherical C60 molecules, and nanoparticles. Antimicrobial PDT has been suggested as an efficient alternative approach to eliminate biofilms. PDT has multiple targets that not only efficiently kill microbial cells, but at the same time weaken the ROS and degrade the matrix structure and the EPS by attacking numerous biomolecules [66]. PTT is another physical approach to eliminate biofilms and interior microorganisms. Under laser illumination, photothermal agents such as metallic, polymeric, and carbon-based nanoparticles can generate local hyperthermia to kill bacteria [67]. PTT triggered by near-infrared irradiation can penetrate the biofilm deeply while causing little damage to surrounding areas, and it can also fight against pathogenic microorganisms by damaging the membrane permeability and metabolic signals, denaturing proteins/enzymes, and inducing bacterial death [68].

Most of the antibiofilm approaches belong to chemical methods. Among the chemical strategies, enzyme degradation is a promising method to decompose biofilms. Enzymes that can degrade these components are expected to be useful to disrupt biofilms and improve the delivery of antibacterial agents. Extracellular DNA is an important component of biofilms and creates a robust barrier. DNase has been proved to be effective in ameliorating antibiotic susceptibility against biofilm-related infections [69]. DNase hydrolyzes the extracellular DNA, which is responsible for cell adhesion and strength. The antibiofilm mechanism of DNase breaks the phosphodiester bond next to the pyrimidine nucleotides in the DNA strands [70]. Proteins are a key part of EPS. Biofilm-associated proteins can be another potential target for antibiofilm management [71]. Proteinase K is a serine protease that cleaves the C-terminal peptide bond for protein digestion. Proteinaceous adhesion during the EPS adherence on the surface can be inhibited by proteinase K [72]. Polysaccharide-degrading enzymes, including dispersin B, dextranase, and mutanase, disintegrate the biofilm matrix via polysaccharide degradation. Dispersin B is a biofilmreleasing enzyme from *Aggregatibacter actinomycetemcomitans* and has been used to eliminate biofilms in pig skin infected by *S. epidermidis* and *S. aureus* [73]. Lysostaphin is a glycylglycine endopeptidase bacteriocin that cleaves the pentaglycine cross-bridge in Staphylococcal peptidoglycan for disorganization of the biofilm matrix [74].

Adhesin production inhibitors and adhesin-binding antibodies can be developed to interfere with microbial binding to the host surface. Mannosides target the bacterial adhesin of FimH to prevent infection by reducing bacterial colonization via adhesion suppression [75]. LL-37 is a cathelicidin-derived broad spectrum antimicrobial peptide that has been investigated to restrain biofilms by preventing bacterial attachment to the surface [76]. LL-37 also displays broad spectrum antibacterial activities and immunomodulatory functions. Oritavancin is another antimicrobial peptide that can suppress bacterial attachment to the surface. It is a semi-synthetic lipoglycopeptide used for treating infections caused by biofilms of MRSA and vancomycin-resistant *S. aureus* (VRSA) [77]. Oritavancin has a structure related to vancomycin, but it shows less toxicity than other lipoglycopeptides, such as telavancin and vancomycin [12]. Antimicrobial lipids, including free fatty acids and monoglycerides, are categorized as single-chain lipid amphiphiles. Due to their amphiphilic nature, these lipids act like surfactants to kill microbes by the mechanisms of increased membrane permeability, enhanced cell lysis, microbial enzyme inhibition, and electron transport chain disruption [78]. Antibacterial lipids can even be used as antibiofilm agents. Glycerol monolaurate is an example of an antimicrobial lipid used to eliminate biofilm structures [79]. The two free fatty acids, docosahexaenoic acid and eicosapentaenoic acid, have been found to display the ability against biofilms developed by *Porphyromonas gingivalis* and *Fusobacterium nucleatum* [80]. Quaternary ammonium compounds have similar structures to antimicrobial peptides, with amphiphilic properties that disarrange the biomass matrix. The core structure of quaternary ammonium compounds comprises a hydrophilic ammonium moiety and a lipophilic alkyl chain to cause bacterial lysis and membrane leakage [81].

Quorum sensing (QS) is a type of cell-to-cell communication that is reliant on signaling molecules known as autoinducers. Autoinducers are produced by bacteria to increase cell density. QS depends upon a sequence of events: signal production, detection, and gene activation. QS has a vital role in the regulation of biofilm formation [82]. QS needs the binding of a signaling molecule to a corresponding transcriptional regulator, which activates the downstream transcription of selected targets [83]. The effect of QS in biofilm establishment offers a strategy to develop novel therapeutics. The inhibition of QS is based on the repression of signal generation, blockading the signal receptors, and interfering with the QS signal. Pathogens are not killed by QS inhibitors. The suppression of the *agr* QS system leads to more bacterial adherence due to biofilm formation, whereas the treatment by autoinducing peptides reactivates the *agr* QS in the biofilm to disassemble the biofilm [9]. Hamamelitannin is a non-peptide compound of the QS inhibitor RNAIII-inhibiting peptide that can reduce *S. aureus* attachment, resulting in the failure of biofilm formation [84]. Antibodies against *S. aureus* QS peptide AP4 have also been suggested to inhibit biofilms in mouse abscess infection models [85]. The QS autoinducer AI-2 functions as a chemorepellent by regulation of the spatial organization of biofilm cells. Exogenous AI-2 treatment on a biofilm results in a reduction in the proportion of adherent bacteria and dispersal [86]. Table 1 summarizes the profiles for antibiofilm treatments using different strategies.

## 5. Different Types of Nanoparticles for Biofilm Eradication

### 5.1. The Antibiofilm Mechanisms of Nanoparticles

Most antibacterial agents have difficulty penetrating through the EPS matrix produced by a biofilm. One of the efforts to resolve this drawback is the intervention of nanoparticles [87]. Nanosystems with intrinsic antimicrobial potential can act as biofilm-targeting agents. These nanoparticles are primarily inorganic materials such as metal oxide nanoparticles. Owing to their flexible structures and controlled drug release, some nanoparticles (especially organic types) function as antibiotic delivery carriers for biofilm eradication. The entrapment of antibiotics in nanocarriers provides drug protection during the delivery process, thereby enhancing and prolonging the antimicrobial efficacy. The interactions between biofilm and nanoparticles are regarded as having three stages: nanoparticle transfer in the biofilm vicinity, attachment to the biofilm surface, and migration into the biofilm [88]. The level of biofilm–nanoparticle interaction depends on the physicochemical properties of the EPS, the nanoparticles, and the environment around the biofilm. The interactions between biofilm and nanoparticles are mainly determined by the electrostatic force. Both the surface charge of the nanoparticles and that of the biofilm matrix regulate the interaction. The presence of uronic acid or pyruvate with the functional groups of carboxylic acid and residual phosphate or sulfate contributes to the polyionic biomass. This negatively-charged matrix facilely interacts with cationic nanoparticles through electrostatic attraction [89]. Once the nanoparticles are deposited in the biofilm, they distribute and diffuse into the biofilm through the EPS matrix. The diffusion of nanoparticles inside the biofilm depends on the pore size of the matrix, the presence of aqueous channels, the environmental lipophilicity, and the charge of the EPS and nanoparticles [88]. There are different ion concentrations in the aqueous pores of biofilms. The penetration of nanoparticles into the biofilm is determined by the ion composition and concentration of the nanoparticle solution.

**Figure 3:**
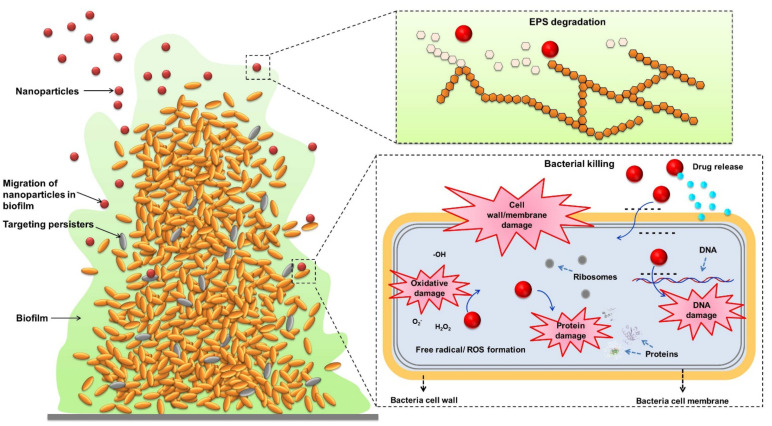
The mechanisms antibiofilm nanoparticles use for eradicating pathogenic microorganisms.

After the nanoparticles’ distribution in the matrix, antimicrobial nanosystems can further kill pathogens. Nanoparticles present efficient antimicrobial activity because of their large total surface areas, providing close contact with microbes. Nanoparticles attach to the bacterial membrane and penetrate it. After penetration into the bacterial cytoplasm, the nanoparticles can kill microbes via protein function inhibition, DNA damage, translation disturbance, and/or transcription dysregulation [90]. Nanoparticle treatment under external stimulation, such as pH, light, and magnetic fields, is exploited to synergize the antibiofilm activity. These responsive nanoparticles are usually metallic nanomaterials [91]. The rich surface chemistry and the nanoscale dimensions have led to the promotion of nanoparticle transport into biofilms and the targeting of microbes when the stimuli are activated. In addition, the non-specific biofilm damage caused by heat or physical force allows for a wide range of biofilms to be targeted [92]. The antibiofilm mechanisms of nanoparticles are illustrated in Figure 3.

### 5.2. Metallic Nanoparticles

**Figure 4:**
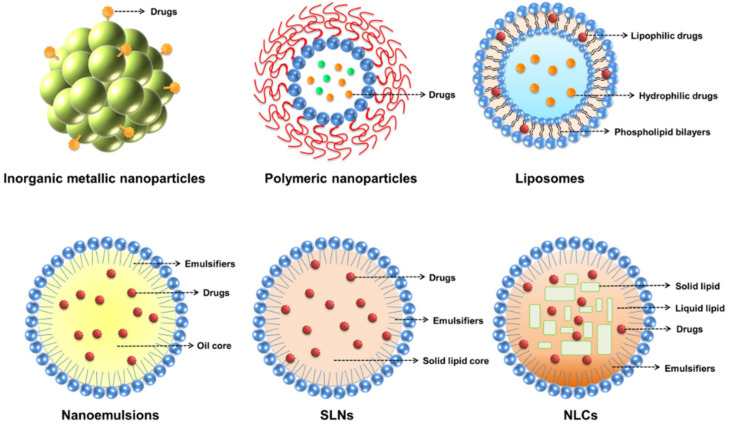
The different types of nanoparticles for inhibiting biofilms.

There are several types of nanoparticles used for the treatment of biofilms (Figure 4). A major type of nanoparticle with intrinsic antibiofilm activity is inorganic metal-based nanoparticles. Metallic nanoparticles are basically rigid particles made of various materials, such as metals (e.g., iron, gold, and silver) and metal oxides (e.g., titanium dioxide, zinc oxide, and iron oxide). Metallic nanoparticles have a broad range of potential applications in microbiology [93]. Specified metallic nanoparticles can release ions to target EPS or microorganisms, and some can interact with EPS as a result of surface functional groups or charge interaction [94]. In general, the main antimicrobial mechanisms of metallic nanoparticles are mechanical membrane damage by electrostatic interaction, oxidative stress as a result of ROS generation, and interference with protein function as a result of metal ion release [95]. Silver nanoparticles are among the most investigated metal-based nanomaterials due to their excellent antimicrobial and antibiofilm activities [96]. The antimicrobial properties of silver nanoparticles are mainly attributed to the direct damage to the bacterial membrane and the continuous ion release. Some metallic nanoparticles are capable of reducing bacterial adhesion on the surface to retard biofilm formation. Zinc ions released from zinc oxide nanoparticles suppress the enzymatic activity of the DapE protein involved in peptidoglycan synthesis, leading to the failure of initial biofilm development [97]. Exposure to metal oxide nanoparticles such as titanium dioxide nanosystems can destroy the biofilm, primarily due to the ROS and lipid oxidation production [98]. In addition to passive electrostatic interaction with the biofilm, metallic nanoparticles can deeply penetrate into the matrix under a magnetic field. In this case, nanoparticles show mechanical activity on the biofilm to destroy the EPS architecture [99]. Other stimuli-assisted antibiofilm metal nanosystems include PDT and PTT. These strategies are based on light or heat-induced biofilm degradation. For instance, gold nanoparticles can receive a high-fluence laser pulse to evoke rapid water evaporation and produce nanobubbles. These nanobubbles generate gaps in the biofilm that facilitate nanoparticle or antibiotic diffusion [96]. Metallic nanoparticles are extensively used topically on the skin to accelerate percutaneous absorption [100]. Topically applied metal-based nanoparticles are especially feasible for treating the biofilms produced in skin infections.

### 5.3. Polymer Nanoparticles

The special features of polymeric nanoparticles are their controllable properties tailored for a particular cargo and to the appropriate size for tissue penetration via passive or active targeting, specific cellular trafficking, and the effortless modulation of drug delivery by sophisticated material engineering. Polymer materials, such as polystyrene, polyvinyl alcohol (PVA), polylactic acid (PLA), polyglycolic acid, poly(lactic-*co*-glycolic) acid (PLGA), poly(ε-caprolactone) (PCL), polyethyleneimine (PEI), poly(acrylic acid) (PAA), poly(glutamic acid) (PGA), and cellulose, have been employed for the preparation of polymeric nanoparticles with the aim of drug delivery or therapeutic applications [101]. Polymer-based nanoparticles can provide physicochemical protection for drugs, proteins, and genes, leading to prolonged biological life and subsequent concentration on the nidus. Polymeric nanocarriers can entrap multiple drugs and hence facilitate synergic therapy. These drugs should be physically loaded or covalently conjugated to the nanostructures [102]. Antibiotic-loaded polymeric nanocarriers are largely applied for eliminating biofilm infections [103]. Polymeric nanosystems have been reported to have inherent antibiofilm effects. These nanoparticles work through mechanisms that involve electrostatic interactions with EPS having negative charges on their outer layers. For example, chitosan polymers are a natural aminopolysaccharide showing antibacterial and antibiofilm activities [104]. Polymeric nanoparticles could be used as potential topical nanocarriers to mask the physicochemical properties of drugs and improve skin delivery. Polymers have the advantages of low toxicity and high biocompatibility during drug delivery. The most well-known mechanisms of the skin permeation enhancement of dendrimers (one of the polymeric nanosystems) are their interactions with skin lipids and the denaturation of keratins, which allow greater transcellular permeation of the drugs. Additionally, they can modify the physicochemical properties of actives and enhance skin partitioning and drug flux [105]. Cutaneous targeting can be achieved through optimization of the particle size, surface charge, and functionalities on polymeric nanoparticles, with minimized skin irritation and other adverse reactions.

### 5.4. Lipid Nanoparticles

Liposomes, nanoemulsions, solid lipid nanoparticles (SLNs), and nanostructured lipid carriers (NLCs) can be classified as lipid-based nanoparticles with a soft feature. Lipid nanoparticles are some of the most promising formulations for drug and gene delivery or for targeting the nidus due to their adaptable physicochemical properties, well-established safety profiles, and the ease of scaling up [106]. Lipid nanocarriers represent a superior alternative to metallic or polymeric nanoparticles, as they are usually composed of USFDA-approved lipid materials. Lipid-based drug-delivery nanocarriers have been extensively used in topical skin applications due to their high drug encapsulation, biocompatibility, increased skin permeation, follicular accumulation, and cutaneous targeting [107]. Liposomes are the most focused-on lipid-based nanoformulations in commercialization and clinical studies because of their possible industrial scale-up, biocompatibility, low toxicity, and capacity to entrap both lipophilic and hydrophilic actives [108]. The structure of a liposome consists of a spherical lipid vesicle composed of phospholipid bilayers. Liposomes have been reported to preferentially adsorb onto the biofilm surface and then penetrate the EPS to inhibit bacterial growth [109]. Due to the affinity of liposomal phospholipid bilayers with biofilms, the liposomes are allowed to fuse with the biomass and the bacterial membrane [110]. Nanoemulsions are another case of antibiofilm lipid-based nanocarriers. Nanoemulsions are isotropic and thermodynamically-stable nanosystems consisting of oil, water, and emulsifiers. They provide significant potential as functional additives in cosmetics, topical drug delivery, and pharmaceutical products. Nanoemulsions are highly beneficial for disassembling biofilms due to their proficient penetration into porous matrices and close contact with the biofilm surface, thereby allowing a high concentration of antibacterial agents [111]. The lipophilic nature of nanoemulsions can produce interaction with the EPS, leading to the disruption and disengagement of the lipid layer. It is possible to mix lipids and polymers to fabricate lipid-polymer nanohybrids. Lipid-polymer nanoparticles consisting of biocompatible lipids and polymers can act as ideal drug delivery carriers by combining the advantages of lipid-based and polymer-based nanosystems [112]. It has been reported that the use of lipid-polymer nanohybrids carrying linezolid can improve the treatment of MRSA infections inside bone cells and biofilms [113].

## 6. Topically Applying Nanoparticles to Treat Cutaneous Biofilms

### 6.1. Metallic Nanoparticles

Most metallic nanoparticles are inorganic forms with a tiny diameter (<20 nm). Metallic nanoparticles offer a platform for topical administration to treat infected biofilms. Due to their ultrafine size, magnetic nature, and easy functionalization, metal oxide nanoparticles have emerged as promising candidates for antibiofilm application. Richter et al. [114] found that colloidal silver with different shapes could disrupt the biofilms developed by *P. aeruginosa*, *S. aureus*, and MRSA. The sizes of the quasi-spherical, cubic, and star-shaped nanoparticles were 40, 70, and 140 nm, respectively. The quasi-spherical nanoparticles revealed lower cytotoxicity against airway epithelial cell line *NuLi-*1** than the others. The in vitro antibiofilm capability evaluated by a resazurin assay demonstrated that the quasi-spherical nanoparticles could eliminate more than 96% of the biofilms of *P. aeruginosa*, *S. aureus*, and MRSA. The in vivo efficacy was tested in an infection model of *Caenorhabditis elegans*. The survival rate of *S. aureus*-infected *C. elegans* was 72%, and this rate increased to 89% after the topical treatment of quasi-spherical nanoparticles. The bacterial CFU was decreased from 3.3 × 10^4^ to 4.5 × 10^3^ after the nanoparticle intervention. McLaughlin et al. [115] developed sprayable silver nanoparticles for treating skin wound-induced biofilms. The nanoparticles were coated with antimicrobial peptide LL-37 and then combined with collagen to form a stable film once sprayed. The silver nanoparticle size was increased from 4 to 750 nm after this decoration. A significant eight-log reduction of *P. aeruginosa* colonies in the biofilm was found after the treatment of the spray. The biofilm treated with the nanoparticles disappeared after one hour. The LL-37-coated silver nanoparticles doubled the silver deposition on the skin wound compared to the formulation without LL-37.

Lazurko et al. [116] further engineered sprayable silver nanoparticles coated by short peptide CLKRS for improving biofilm-associated wound healing. To support skin regeneration, a thermoresponsive collagen matrix containing a full-thickness microscopic skin tissue column (MSTC) was used as the vehicle for the nanoparticles. MSTC is a new therapy that harvests micrometer-sized skin for skin regeneration without scarring [117]. The spray application was sufficient to confer a four-log reduction in the proliferation of *P. aeruginosa* and *S. aureus*. The nanoparticles also prevented the growth of biofilms by both bacteria. A full-thickness open wound model in diabetic mice was used to test the in vivo antibiofilm effect. A significant decrease in the number of surviving *P. aeruginosa* was detected following the CLKRS-coated nanoparticle treatment on the wound site as compared to the untreated control. Silver nanoparticles were loaded in thermoreversible Pluronic F-127 hydrogels for antibiofilm treatment of a skin wound [118]. The hydrodynamic size of the silver nanoparticles used in this study was 9 nm. The log reductions of *S. aureus* in the biofilm were 0.33, 2.0, and 5.7 after treatment using the silver nanoparticle suspension, the silver nanoparticle-loaded hydrogel, and a commercial silver sulfadiazine cream, respectively. The human fibroblast viability with all gel formulations was >95%, in contrast to the silver sulfadiazine cream, which showed a viability of only 18%. Alginate wound dressings offer a moist microenvironment to limit bacterial infection and enhance wound healing [119]. Ambrogi et al. [120] prepared an alginate film containing silica-supported silver nanoparticles as a wound dressing. Silver nanoparticles sized 8−20 nm were uniformly dispersed and grown on a silica shell. The silver nanoparticle-loaded film showed significant antibiofilm activity against *P. aeruginosa* and *S. aureus* by the determination of crystal violet in a static biofilm assay. This film demonstrated no cytotoxicity towards human fibroblasts and keratinocytes, thereby suggesting its use is safe.

Copper oxide is also valuable for removing biofilms. Copper ions can be incorporated into mesoporous glass nanoparticles due to their excellent textural characteristic [121]. This nanosystem was proposed to treat chronic wounds infected by *P. aeruginosa* and *S. aureus*. The developed nanoparticles evidenced a large surface area (740 m^2^/g), uniform pores with a diameter of 4 nm, and a particulate size of 100−150 nm. The exposure of bacteria to the nanoparticles destroyed the biofilm when considering the biomass and biofilm metabolic activity, with a greater inhibition on *P. aeruginosa* than *S. aureus*. The nanosystem revealed similar efficacy against *S. aureus* biofilm with commercial Acticoat Flex 3 (a silver-coated dressing) in an engineered tissue infection skin model. Copper was combined with silver to form bimetallic nanoparticles for antibiofilm treatment [122]. The average diameter of the nanoparticles was about 7 nm. The bimetallic nanoparticles were coated on a 2D grapheme oxide sheet to provide a sustained metal release and regulate the oxidation of the metals. The nanosheet successfully removed the *P. aeruginosa* biofilm in a microchannel with a dynamic flow. In the untreated control group, the size of the biofilm area was increased by 800% after 72 h. This region was reduced to only 30% by the nanosheet as compared to the antibiotic Doripenem (260%). In the in vivo wounded mouse model infected by *P. aeruginosa*, the closure of the PBS-treated wound was impaired after seven days. The nanosheet effectively reduced the biofilm-associated wound closure delay.

Multivalent aminosaccharide-based gold nanoparticles are efficient at killing MRSA due to the similarity between aminosaccharides and bacterial peptidoglycans [123]. Yang et al. [124] used aminosaccharide-based gold nanoparticles to test the in vivo antibiofilm activity against MRSA-infected skin wounds. The fluorescein diacetate test was employed to detect *S. aureus* cells in a biofilm. More than 93% of the bacteria in the biofilm were dead after treatment with the gold nanoparticles. In the in vivo MRSA-infected skin wound of a rat, the size of the wound was reduced to 68% by commercial silver nanoparticles as compared to the control after a seven-day application. A further decrease was observed using the aminosaccharide-based gold nanoparticles (<40%). Raghuwanshi et al. [125] combined *Woodfordia fruticosa* extract with gold nanoparticles to prevent microbial biofilms and enhance skin wound healing. Fresh flowers of *W. fruticosa* were used for rapid wound healing [126]. A synthesized nanosystem with a diameter of 10−20 nm was utilized to fabricate Carbopol 934 hydrogel for topical use. Scanning electron microscopic morphology revealed that the *C. albicans* and *C. neoformans* biofilms were disrupted, scattered, and distorted by the nanoparticles. The fungi inside the biofilms damaged hyphae and a ruptured yeast form. In the rat wound closure test, the maximum epithelialization period of the nanoparticle-loaded hydrogel treatment group was 15.5 days. This recovery duration was shorter than that of the untreated control (23.2 days) and hydrogel (21.8 days).

Zinc oxide nanoparticles were prepared using a biopolymer starch as the capping agent to assess the antibiofilm effect [127]. The mean particle size of the developed nanoparticles was approximately 500 nm. The confocal microscopic images of the *P. aeruginosa* and *S. aureus* biofilms depicted a disintegrated architecture after nanoparticle intervention. *S. aureus* was injected through an intradermal route into mice to develop a skin infection model. The topical application of zinc oxide nanoparticles reduced the bacterial CFU by 118-fold as compared to the control. This reduction could be increased to 165-fold by using smaller-sized nanoparticles (<50 nm). Zinc oxide nanoparticles were coated onto polyester-nylon wound dressings to provide a topical application with improved antibiofilm activity [128]. The shape of the zinc oxide nanoparticles was quasi-spherical and had an average size of 40 nm. The nanoparticle-containing dressing could reduce the viability of *S. aureus* and *E. faecalis* through the cell membrane integrity loss. The dressing was beneficial for suppressing biofilm growth for a wide range of bacteria, including *P. aeruginosa*, *S. aureus*, *E. faecalis*, and *E. coli*.

Metal oxide nanoparticles can be responsive to external stimulation for eradicating biofilms. An example is the induction of hyperthermia by a magnetic field. Kim et al. [129] evaluated the effect of antimicrobial magnetic thermotherapy on biofilm disorganization. An electromagnetic generator was designed to deliver high specific loss power to the ferromagnetic Fe_3_O_4_ nanoparticles. Moreover, anti-protein-A antibodies were attached to the nanoparticulate surface to facilitate specific targeting of the surface coat of *S. aureus*. The estimated maximum binding efficiency of the protein-A-targeted nanoparticles to the biofilm was about 50%. A three-log elimination of *S. aureus* CFU in the biofilm was achieved in the presence of a magnetic field (40 kA/m). The inoculation of the nanoparticles into the *S. aureus*-infected open wound did not cause any change in the bacterial burden in the absence of hyperthermia. The anti-protein-A targeting enhanced the bacterial inactivation by 80%. The application of hyperthermia maintained a low *S. aureus* load in the wound. The enhanced inactivation by the targeted nanoparticles correlated with increased wound closure. The skin temperature at the wound margin increased to a maximum of 43 °C, which was tolerable to the skin. PTT could impair biofilm structures through physical heat [130]. Near-infrared light within the wavelength range of 700−1100 nm was efficient for tissue penetration with minimal damage to healthy tissue. Hu et al. [131] developed gold nanoparticles that were responsive to both near-infrared irradiation and the acidic microenvironment of biofilms for the photothermal ablation of MRSA biofilms. The pH-responsive property was generated by incorporating a zwitterionic self-assembled monolayer of 11-mercaptoundecanoic acid in the nanoparticles. The surface charge of the nanosystem became positive when the environmental pH was reduced to 5.5, which promoted electrostatic attraction to the MRSA. Once irradiated by near-infrared light, the nanoparticle-treated biofilm elevated the temperature to 60 °C. Subsequently, most of the MRSA in the biofilm were killed. The nanoparticles were directly injected in a subcutaneous abscess produced by local MRSA infection in rabbits. After the irradiation, the MRSA burden in the abscess was decreased from 120 × 10^6^ to 30 × 10^6^ CFU/mL, and there was no sign of inflammation.

Bismuth nanoparticles have successfully acted as photothermal agents to elicit PTT. However, the instability and oxidation of bismuth under physiological conditions have limited their practical application [132]. The incorporation of bismuth nanoparticles onto mesoporous silica could improve their stability and increase the photothermal conversion effect. Cao et al. [133] fabricated mesoporous silica-supported bismuth-silver nanoparticles as a photothermal agent to eliminate MRSA biofilms. This nanoformulation exhibited good photothermal stability and increased the temperature from 24 to 46 °C under near-infrared radiation (1 W/cm^2^ for 15 min). The hyperthermia induced by the combined nanoparticle and near-infrared irradiation obliterated the MRSA biofilm and caused a 70% reduction in the biomass, demonstrating a better effect than that based on nanoparticles without silver (27%) and nanoparticles without irradiation (31%). The antibiofilm potential was appraised in a mouse model with a subcutaneous MRSA infection. The in vivo data indicated that 95% of the MRSA in the abscess were killed, and the abscess ablation was ameliorated after the PTT. Nanozymes are nanomaterials that mimic the activity of enzymes for biomedical use. Nanozymes that simulate peroxidase can convert hydrogen peroxide into bacterial free radicals [134]. Xu et al. [135] designed a photothermal nanoplatform based on the entrapment of tungsten sulfide quantum dots as the nanozymes and vancomycin as the antibacterial agent in liposomes. The hydrodynamic diameter of the quantum dots was around 11 nm. The vesicle size of the liposomes incorporated with quantum dots and vancomycin was 146 nm. The increased temperature caused by the photothermal effect promoted the oxidase-like activity of the nanosystem to completely disrupt the vancomycin-intermediate *S. aureus* (VISA) biofilm because of the deep penetration. Mice bearing VISA-infected skin abscesses manifested rapid skin recovery, with scars vanishing after 12 days by the topical application of PTT. The temperature of the abscess area increased from 25 to 45 °C after near-infrared exposure (1 W/cm^2^ for 5 min) in the nanosystem. The PDT induced by the combined photosensitizer and light was found to be active in biofilm clearance [136]. Sherwani et al. [137] explored the photodynamic effect of gold nanoparticles conjugated with methylene blue and/or toluidine blue O as a photosensitizer against *C. albicans* biofilms. The nanoparticles conjugated with the combined methylene blue and toluidine blue O showed a fungal viability of 20% in mature biofilms under PDT. This effect was greater than that using nanoparticles with only one photosensitizer (a fungal viability of 40−50%). The cutaneous *C. albicans* infection in mice indicated a 50% CFU reduction using nanoparticles with the combined photosensitizers. The PDT manifested a pronounced reduction of the yeast form and hyphal filament of *C. albicans* in the biofilm. The dual stimuli-responsive approach could further synergize the antibiofilm activity as compared to the single stimulus. Xiao et al. [138] designed a photothermal/pH dual stimuli-sensitive metal-organic nanosystem based on the zeolitic imidazolate frameworks-8 (ZIF-8) with antimicrobial activity. ZIF-8 with polydopamine surface decoration was used to entrap vancomycin and construct the stimuli-responsive nanocomposite. The hydrodynamic size of the nanocomposite with and without antibiotics was 176 and 168 nm, respectively. The hyperthermia induced by near-infrared light in conjunction with pH-dependent nanoparticle leakage for vancomycin release enabled control of the drug delivery to eliminate VISA biofilm formation. Crystal violet staining revealed that 76% of the biofilm was eliminated by the PTT induced by the vancomycin-loaded nanoparticles, whereas the biofilm eradication was 60% in the group of nanoparticles without antibiotics. This PTT was applied to an in vivo mouse model with subcutaneous VISA invasion. As compared to the PBS-treated control, the combined near-infrared irradiation and vancomycin-loaded nanoparticles reduced the viable bacterial colony to 1.8%. The PTT also minimized the infiltration of immune cells in the wound site as compared to the control. The profiles of the metallic nanoparticles employed for topical antibiofilm therapy on the skin are summarized in Table 2.

### 6.2. Polymeric Nanoparticles

In the process of developing nanomedicine, some polymers have been broadly explored to prepare nanoparticles because of their excellent biocompatibility, high drug loading, easy manipulation, and low cost [139]. Polymeric nanocarriers can serve as ideal vehicles for antibiotics due to the optimization of the physicochemical properties of the drugs. Chemically-synthesized nanoparticles coated with biocompatible polymers can reduce the toxicity. For instance, polysaccharides from algae are vital for increasing the biocompatibility of metal oxide nanoparticles. El-Deeb et al. [140] biologically synthesized silver nanoparticles using *Arthrospira sp.* polysaccharides to evaluate their antibiofilm effect and safety profile. Scanning electron microscopy provided an estimation of the nanoparticles coated with algal polysaccharides with a diameter of 9.7 nm. Treatment of *P. aeruginosa* with the nanoparticles contributed to the disorganization of the bacterial outer membrane and the reduction of biofilm formation. A 60% inhibition of biofilm formation was detected after nanoparticle treatment using crystal violet analysis. The nanoparticles showed substantial activity to retard *P. aeruginosa* infections in rat skin, resulting in the reduction of infiltrated immune cells and hemorrhagic area number. After the nanoparticles’ application to the wounded skin, the amount of overexpressed COX-2 decreased from 14 to 10 ng/mL. PLGA is commonly used as a biocompatible polymer for nanoparticle fabrication. Zhang et al. [141] employed PLGA to develop polymeric nanoparticles for loading ciprofloxacin with the aim of sustained delivery. Inspired by a delivery system that mimics marine mussels for adhesion, bioadhesive nanoparticles contained in hydrogel were designed to enhance topical antimicrobial delivery. The free ciprofloxacin displayed a burst release from the gel, with a 94% release during the first 12 h. The nanoparticulate inclusion of the antibiotic reduced the release rate, with 88% release after 72 h. The nanoparticles were attached to an *E. coli* biofilm under a flow condition. It was observed that nearly 100% of the bioadhesive nanoparticles remained on the biofilm after the flow, whereas 8% of the nonadhesive nanoparticles were retained. In the study of in vitro antibacterial activity evaluation by inoculating *E. coli* onto a porous polycarbonate membrane, the bacterial growth was increased by an order of four in the untreated control. The nanoparticles could eliminate the bacterial number by 20-fold as compared to the control, suggesting the benefit of sustained drug release and bioadhesive properties for bacterial eradication.

Nitric oxide (NO) is favorable for dispersing biofilms for wound healing [142]. Due to the short half-life of NO in biomasses, Hasan et al. [143] developed polyethylenimine/diazeniumdiolate-doped PLGA nanocomposites with the ability to bind to biofilms for facilitating NO delivery to MRSA-infected skin wounds. Polyethylenimine/diazeniumdiolate was used as the NO donor in this nanosystem. The prepared nanosystem displayed an average diameter of 240 nm. The nanoparticles exhibited a sustained NO release over four days in the simulated wound fluid. The cationic nature of the nanosystem, with a zeta potential of 25 mV, led to the electrostatic attraction with the biofilm matrix. Using crystal violet staining, the PLGA nanoparticles reduced the biomass by 67% after a 24-h treatment. The topically applied nanosystem accelerated the healing of MRSA-infected wounds in diabetic mice, with a 92% wound closure after 12 days. The control group (blank nanoparticles) showed no reduction in wound size. Almost no MRSA load was detectable at day 12 post-injury. Hyaluronic acid (HA) is a natural polymer of disaccharides and is regarded as a drug delivery system due to its biocompatibility and ease of functionalization [144]. HA was employed as the major material to prepare a nanogel loaded with the antibiofilm peptide DJK-5 [145]. The nanogel with a size of 174−194 nm entrapped 33−60% of the peptide. The peptide-loaded nanogel presented bacteriostatic activity against *P. aeruginosa* for five hours and subsequent regrowth after 24 h. The nanogel was further investigated for its antibiofilm activity in a murine *P. aeruginosa* abscess model. The bacterial colony in the abscess treated by the DJK-5-loaded nanogel was four-fold less than that of the nonloaded control. Chitosan and benzalkonium chloride (BKC) were loaded in PLGA nanoparticles to act as antimicrobial agents and promote MRSA-infected skin wound healing [146]. Chitosan is a cationic linear polysaccharide that shows antibiofilm effects [147]. The cationic surfactant BKC has also been extensively used for topical applications to kill bacteria. The data from a crystal violet assay showed a four-fold reduction of BKC-loaded PLGA-chitosan nanoparticles compared to free BZK. The biofilm was diffused and damaged after the treatment of the nanocomposites. In the in vivo MRSA-infected full-thickness wound healing experiment, the bacterial burden reductions were 80% and 56% after topical administration of the nanoparticulate and free BKC, respectively. After 14 days of treatment, the wound healing reached 100% and 82% for the nanosystem and free BKC groups, respectively.

Microneedling is a physical method to enhance drug permeation across the skin. A *microneedle* device is made by arranging hundreds of *microneedles* in arrays on a tiny patch for producing pores on the skin, thereby facilitating drug delivery [148]. Permana et al. [149] presented a combination of bacteria-sensitive nanoparticles and dissolving microneedles of doxycycline for improved biofilm delivery and specific drug skin permeation to the infection area. The nanoparticles were prepared from PLGA and PCL decorated with chitosan. The polymeric nanoparticles showed a size range of 217−263 nm and had a doxycycline encapsulation percentage of 43−53%. The doxycycline release was increased up to seven-fold in the presence of a biofilm. The free drug only killed *P. aeruginosa* and *S. aureus* biofilms by 30−52%, whereas more than 99% of the bacteria were killed by the nanoparticles. The loading of nanoparticles in microneedles could enhance drug transport into excised pig skin, suggesting a higher retention time compared to the needle-free patch. In the ex vivo biofilm model on pig skin, almost all bacteria were eradicated by the nanoparticle-incorporated microneedles, whereas 60% of the bacteria remained on the control patch. Mir et al. [150] further prepared carvacrol-loaded bacteria-responsive PCL nanoparticles and combined them with microneedles. The phenolic monoterpenoid carvacrol was derived from plant essential oils manifesting antibacterial activity against both planktonic and biofilm microorganisms [151]. The carvacrol-loaded nanoparticles resulted in a greater reduction of viable bacteria in the biofilm as compared to the free control. The eradication rate was 88−100% for *S. aureus* and MRSA. The dermatokinetic study demonstrated that the microneedles delivered 8.5 times the amount of carvacrol in the form of nanoparticles compared to the free carvacrol after topical delivery to neonatal pig skin. Following the biofilm infection in the ex vivo pig skin wound model, the combined nanoparticles and microneedles caused >99% inhibition of *S. aureus* and *P. aeruginosa* colonies in the wound. Singh et al. [152] designed pH-sensitive alginate polymer nanoparticles to deliver ciprofloxacin and the QS inhibitor 3-amino-7-chloro-2-nonylquinazolin-4(3*H*)-one to target mature *P. aeruginosa* biofilms. The nanoparticles were engineered to incorporate a pH-responsive linker between the alginate and the QS inhibitor. In this way, the release of the antibiotic and the QS inhibitor was triggered in the low-pH region of the biofilm. Alginate oligosaccharides have been proved to disorganize biofilms and potentiate the antibacterial effect of antibiotics [153]. The produced pH-sensitive nanocomposites displayed a mean size of 179 nm. As tested in the *P. aeruginosa* biofilm model, the concomitant release of both agents from the nanoparticles greatly reduced the bacterial viability in the biofilm as compared to ciprofloxacin alone. An ex vivo model of the *P. aeruginosa* biofilm on pig skin was established to examine the antibiofilm activity. The result showed complete clearance of bacterial load in the infection site due to the nanocomposites, but not in the cases of free ciprofloxacin or the nanosystem containing only the QS inhibitor. The profiles of the polymer-based nanoparticles employed for topical antibiofilm therapy on skin are summarized in Table 3.

### 6.3. Lipid-Based Nanoparticles

Lipid-based nanoparticles are nanosystems that are rich in lipids in the core matrix or the particulate surface. Lipid nanocarriers are biodegradable and biocompatible, and their formulations can be tailored for antibacterial application. Lipid nanoparticles, such as SLNs, NLCs, and nanoemulsions, are feasible as drug nanocarriers due to their lower toxicity compared with polymeric and metallic nanoparticles. The main difference among these lipid nanocarriers is the composition of the inner matrix. SLNs have a matrix consisting of crystalline solid lipids. The core of NLCs is composed of mixed solid and liquid lipids. Nanoemulsions are nanocarriers with neat liquid lipids in their inner core [154]. Liposomes and niosomes are the lipid-based nanovesicles with an aqueous core and lipid shell. The antibiotic tetracycline and anti-inflammatory tretinoin were incorporated in liposomes for acne treatment [155]. The physicochemical characterization revealed that liposomes with a size of 111 nm could successfully encapsulate both drugs, with an entrapment efficiency greater than 80%. The liposomes sustained the release of the drugs within 24 h, with a percentage release of 56% and 58% for tetracycline and tretinoin, respectively. In terms of the *Streptococcus epidermidis* biofilm susceptibility test, an improved effect of biofilm growth inhibition was found by treatment using drug-loaded nanovesicles. Li et al. [156] developed daptomycin-loaded flexible liposomes for increased skin permeation and antibiofilm activity. Sodium cholate was inserted into the phospholipid bilayers of the liposomes to soften the membrane. The deformable nanovesicles were formed to facilely penetrate across the stratum corneum. The mean diameter and daptomycin encapsulation were 55 nm and 88%, respectively. The disrupted *S. aureus* biofilm was visualized by scanning electron microscopy after the treatment of the flexible liposomes. Very few viable microbes were detected in the liposome-treated biofilm, whereas the *S. aureus* population in the untreated control achieved a 10^8^ order of magnitude. The *S. aureus* biofilm was subcutaneously injected into mouse skin to develop an abscess. The biofilm treated with the control group was thickly dotted with viable bacteria, and the topical treatment of liposomes presented only scattered pathogens on the broken biofilm as observed by microscopy.

Chlorhexidine is an antiseptic that is largely used on skin burn wounds to decrease microbial susceptibility [157]. The antibacterial and antibiofilm potential against MRSA-infected burn wounds of chlorhexidine-loaded nanoemulsions have been estimated [158]. The nanoemulsions could disrupt and disperse MRSA biofilms. The dead/live ratio of MRSA in the biofilm was 84% after the nanoemulsion intervention. This ratio was 6.4 times greater than that of the free antiseptic. The MRSA burden in the burn wound site of mice was reduced by 66% or 45% after topical administration of the nanoemulsions or free control, respectively. Lewińska et al. [159] designed nanoemulsions by using N-oxide surfactants as the emulsifiers to enhance the percutaneous absorption and antibiofilm effect against *C. albicans*. In pig ear skin penetration, the nanoemulsions containing a single-head surfactant with a size of 85 nm showed less absorption than those containing a double-head homologue with a size of 78 nm. Nevertheless, both nanoformulations accelerated skin permeation as compared to the free dye control. Curcumin was loaded into nanoemulsions for evaluation of the in vitro antibiofilm effect. The curcumin-loaded nanoemulsions soaked into the wound dressing dramatically suppressed *C. albicans* biofilms with a maximum level of 80%. In contrast, the free curcumin only inhibited the fungal biofilm by 30%. Lin et al. [160] compared the antibiofilm activity between liposomes and nanoemulsions. A cationic surfactant soyaethyl morpholinium ethosulfate (SME) exhibited two roles in liposomes and nanoemulsions: an emulsifier and an antibacterial agent. The mean diameters of the liposomes and nanoemulsions were respectively estimated as 75 and 214 nm. By using confocal microscopy, it was found that the liposomes lessened the MRSA biofilm thickness by 1.6-fold. This reduction could be further enhanced by nanoemulsions to display a 2.4-fold decrease. The mouse skin was infected by MRSA via subcutaneous injection. Both nanosystems were effective in recovering the barrier function of the skin disrupted by MRSA, with the nanoemulsions showing superior recovery. The bacterial CFU for the liposome-treated skin was lowered by about 200-fold as compared to the PBS control. A further CFU reduction was detected by the nanoemulsion group. SME was also incorporated on the shell of NLCs to determine the efficacy in eradicating MRSA biofilms [161]. To achieve better performance, the NLCs were combined with the antibiotic oxacillin to synergize the antibiofilm activity. The combined NLCs and oxacillin diminished the MRSA biofilm thickness from 31 to 13 μm, which was lower as compared to the effect of the NLCs (18 μm) or oxacillin (25 μm) alone. The topical application of NLCs with oxacillin on MRSA abscesses in mouse skin inhibited the microbial colonies by 4-log.

The antibiofilm silver sulfadiazine-loaded SLNs were dispersed in a chitosan hydrogel for the application of skin wound healing [162]. The mean diameter of the prepared nanoformulation was estimated to be about 300 nm. Confocal microscopy was used to detect the live/dead bacteria and confirmed that the SLNs removed 79% of the *P. aeruginosa* biofilm. For further biomass degradation, SLNs combined with DNase I eradicated 97% of the biofilm after a 72-h treatment. The burn wound healing study in rats demonstrated that the combined SLNs and DNase I produced complete wound healing after 21 days. The SLNs alone and marketed silver sulfadiazine cream showed wound area retraction of 95% and 76% after 21 days, respectively. An oleylamine zwitterionic lipid was combined with chitosan to form vancomycin-loaded lipid-polymer nanohybrids for treating MRSA biofilm infections [163]. This lipid had the feature of zwitterionic pH sensitivity. The surface charge of the nanohybrids was switched from negative to positive when the environmental pH was changed from 7.4 to 6. This feature was preferable for nanoparticle interactions with the biofilm in skin infections with an acidic environment. The in vitro biofilm elimination examined by the live/dead ratio manifested a greater biofilm reduction by the nanohybrids compared to the free vancomycin. The intradermal MRSA infection in the mice generated a bacterial burden of 3 × 10^5^ CFU/mL in the injection site. This colony could be reduced by nanohybrids and free vancomycin to 2.5 × 10^4^ and 266 CFU/mL, respectively. The profiles of the lipid-based nanoparticles employed for topical antibiofilm therapy on the skin are summarized in Table 4.

## 7. The Safety of Nanoparticles on Skin

For the development of nanoparticles for topical use, a prerequisite is the confirmation of minimal toxicity or irritation on skin. Thus, a balance between biological efficacy and safety can be achieved. Since most of the materials used for biomedical nanoparticles can be categorized as generally recognized as safe (GRAS), approved by FDA, it is expected that the topically applied nanoparticles for antibiofilm treatment will be tolerable to the skin. The animal study is commonly employed as a model to preliminarily examine the possible skin irritation induced by topical nanoparticles based on the guideline of Organization for Economic Cooperation and Development (OECD TG404) [164]. It is a useful platform for evaluating the possible skin toxicity elicited by nanoparticles. The growing ethical recognition of animal welfare has prompted the replacement of in vitro tests of skin irritation as alternatives. In the recent years, the reconstructed 3D human skin equivalent is largely used for regulatory purpose to examine skin toxicity after nanoparticle treatment. This model uses reconstructed human epidermis obtained from non-transformed epidermal keratinocytes, which mimic the histology, morphology, biochemistry, and physiology of human epidermis [165]. The guidelines of 3D skin equivalent for toxicity assay are based on OECD TG439 and TG431. The nanoparticle types generally used for antibiofilm treatment include metal, polymer, and lipid-based nanosystems. A concern about the use of heavy metals as the materials in nanoparticles is the possibility of toxicity induction. However, the metallic nanoparticles for topical application are usually safe for skin. The 3D epidermis model revealed that the nanoparticles containing aluminum oxide, titanium oxide, zinc oxide, and silver do not irritate the skin [166,167,168].

The most widely-used polymer material in polymeric nanoparticles is PLGA. In human safety test, PLGA nanoparticles did not cause any erythema and adverse reaction on volunteers’ skin [169]. The lipids used for fabricating lipid-based nanoparticles are mainly the natural oils. These lipids including soybean oil, coconut oil, sesame oil, linseed oil, and grape seed oil are already verified to be biodegradable and biocompatible. The topically applied NLCs are reported to be safe to cause no skin irritation in human [170]. The lipid nanoparticles made with linseed oil is safe for skin application because the nanoparticles were not irritant, sensitizing, and comedogenic on skin [171]. Liposomes are attractive as the antibacterial nanovesicles because of the similarity to biological or cellular membranes. An earlier study [172] demonstrates that the liposomes showed a very low acute irritancy on human skin in clinical trial. The skin toxicity of antimicrobial agents is even reduced by liposomes due to the retardation of the direct contact between skin and antibacterial drugs [173]. It should be cautious that the nanoparticles can cause allergic response on skin by the immunomodulatory effect. This effect is based on the fact that some nanomaterials penetrate into the deep strata of the skin [174]. The main aim of the antibiofilm nanosystems should reside on the skin’s surface for eliminating biofilm or bacteria with less permeation into the skin. This superficial location on the skin’s surface without deep penetration may lead to the lessening of irritation response elicited by the topically applied nanoparticles. The positively charged materials such as chitosan and BKC are largely employed as the antimicrobial agents loaded in the nanoparticulate surface to kill the pathogens. The cationic nanoparticles show the capability to damage skin architecture by disrupting the keratinocyte structure [175]. The examination of possible skin irritation is needed for the development of antibacterial nanoparticles bearing positive charges.

## 8. Conclusions

Antibacterial therapy usually exhibits an incomplete response to biofilm infections in the skin. A major reason is the biofilm-mediated tolerance, which prompts the failure of antibiotic management. The opportunistic microbes on the skin are notorious due to their biofilm formation, which reduces the susceptibility to antibacterial treatment. Currently, there is no clinically approved agent targeting bacterial biofilms. The investigation of delivery carriers that can target biofilms and disassemble the EPS is therefore of importance. Considering the efficiency of biofilm penetration and elimination, the introduction of nanoparticles could be a promising solution for treating biofilm infections on the skin. In this review, the recent advances of nanoformulations for enhanced antibiofilm treatment were summarized. The selection of nanoparticle type is important for the delivery of antibiotics to display maximum activity and minimum side effects. The use of nanoparticles is considered an efficient strategy for biofilm targeting because of their numerous advantages over conventional formulations, including improved stability, sustained drug release, targeted capability, environmental responsiveness, and increased bioavailability. Some issues, such as solubility, drug resistance, and epithelium permeation, could also be resolved by the nanomedicine approach. Regarding future applications, effort should be paid to connecting the gap between laboratory investigation and clinical use. Most studies on topical antibiofilm nanoparticles have been conducted using animal models, and clinical studies have been lacking until now. Another concern is large-scale manufacturing for the market. The topical use of antibiofilm nanoparticles focuses on infected wound healing. There is a need to extend the application of these nanosystems to skin diseases such as AD, acne, and folliculitis. Nevertheless, the availability of previous FDA-approved nanomedicines has encouraged the potential of antimicrobial nanoparticles for clinical use.

## Figures and Tables

**Table 1:** The treatment strategies of biofilm eradication.

The Strategy	Classification	The Mechanism of Eradication
Water spray	Mechanic force	The physical removal of biofilm by local delivery of mechanic forces
Water-based jet	Mechanic force	The physical removal of biofilm by local delivery of mechanic forces
Photodynamic therapy	Physical method	The combination of specific light irradiation and photosensitizers to produce oxidative stress
Photothermal therapy	Physical method	The combination of near-infrared irradiation and photothermal agents to produce local hyperthermia
DNase	Enzymatic degradation	Hydrolysis of the extracellular DNA
Proteinase K	Enzymatic degradation	Cleavage of the C-terminal peptide bond for protein digestion
Dispersin B	Enzymatic degradation	Biofilm-releasing enzyme from *Aggregatibacter actinomycetecomitans* to eliminate biofilm
Lysostaphin	Enzymatic degradation	Cleavage of pentaglycine cross-bridge in peptidoglycan
Mannosides	Adhesin inhibition	Target to bacterial adhesin FimH for prevent bacterial binding to surface
LL-37	Antimicrobial peptide	Prevention of bacterial attachment to surface
Oritavancin	Antimicrobial peptide	Prevention of bacterial attachment to surface
Glycerol monolaurate	Antimicrobial lipid	The amphiphile nature to disrupt biofilm structure
Free fatty acids	Antimicrobial lipid	The amphiphile nature to disrupt biofilm structure
Quaternary ammonium compounds	Surfactant	The amphiphile nature to disrupt biofilm structure
Autoinducing peptides	QS inhibitor	Reactivates *agr* QS in biofilm to disassemble biofilm
Hammelitannin	QS inhibitor	QS inhibitor RNAIII-inhibiting peptide to reduce *S. aureus* attachment
AP4 antibody	QS inhibitor	Biofilm inhibition in mouse abscess infection model
AI-2	QS inhibitor	Reduction of proportion of adherent bacteria and dispersal

AI, autoinducer; QS, quorum sensing.

**Table 2:** The application of metallic nanoparticles for antibiofilm treatment.

Nanoparticle Type	Average Size	Infection Model	The Microorganisms Tested	Antibiofilm Efficacy	Reference
Silver	40, 70, or 140 nm	*C. elegans*	*P. aeruginosa*, *S. aureus*, and MRSA	Biofilm elimination by >96%	Richter et al. [114]
Silver	750 nm	Full-thickness skin wound in mice	*P. aeruginosa*	A 8-log reduction of bacterial colony in biofilm	McLaughlin et al. [115]
Silver	5−12 nm	Full-thickness skin wound in diabetic mice	*P. aeruginosa* and *S. aureus*	Bacterial number reduction in skin open wound	Lazurko et al. [116]
Silver	9 nm	In vitro drip flow reactor model	*P. aeruginosa* and *S. aureus*	A 2-log reduction of bacterial colony in biofilm	Alvarado-Gomez et al. [118]
Silver	8−20 nm	In vitro static biofilm assay	*P. aeruginosa* and *S. aureus*	Elimination of biomass determined by crystal violet assay	Ambrogi et al. [120]
Copper	100−150 nm	3D tissue engineered infection skin model	*P. aeruginosa* and *S. aureus*	Elimination of biomass and biofilm metabolic activity	Paterson et al. [121]
Copper and silver	7 nm	Full-thickness skin wound in mice	*P. aeruginosa*	Biofilm area reduction by 70%	Jang et al. [122]
Gold	4 nm	MRSA-infected skin wound in rats	*S. aureus* and MRSA	A 93% killing of bacterial number in biofilm	Yang et al. [124]
Gold	10−20 nm	Full-thickness skin wound in rats	*C. albicans* and *C. neoformans*	The biofilm is disrupted, scattered, and distorted	Raghuwanshi et al. [125]
Zinc	50 and 500 nm	Intradermal injection of bacteria in mice	*S. aureus*	The biofilm is disintegrated	Pati et al. [127]
Zinc	40 nm	In vitro static biofilm assay	*P. aeruginosa*, *S. aureus*, *E. faecalis*, and *E. coli*	Biofilm growth suppression	Rayyif et al. [128]
Ferrous oxide with hyperthermia	About 100 nm	*S. aureus*-infected skin wound in mice	*S. aureus*	A 3-log reduction of bacterial conoly in biofilm	Kim et al. [129]
Gold with PPT	14 nm	MRSA-induced abscess in rabbits	MRSA	Most of MRSA in the biofilm is killed	Hu et al. [131]
Bismuth-silver with PPT	15 nm	MRSA-induced abscess in mice	MRSA	Biofilm elimination by 70%	Cao et al. [133]
Quantum dot with PTT	11 nm	VISA-infected skin abscess in mice	VISA	Complete disruption of biofilm	Xu et al. [135]
Gold with PDT	10−20 nm	Cutaneous infection in mice	*C. albicans*	A 80% killing of fungal number in biofilm	Sherwani et al. [137]
Zeolite with PTT	About 170 nm	VISA-infected skin abscess in mice	VISA	Biofilm elimination by 76%	Xiao et al. [138]

MRSA, methicillin-resistant *Staphylococcus aureus*; PDT, photodynamic therapy; PPT, photothermal therapy; VISA, vancomycin-intermediate *S. aureus*.

**Table 3:** The application of polymeric nanoparticles for antibiofilm treatment.

Nanoparticle Type	Average Size	Infection Model	The Microorganisms Tested	Antibiofilm Efficacy	Reference
Algal polysaccharides	About 10 nm	*P. aeruginosa* infection in rat skin	*P. aeruginosa*, *S. aureus*, *S. mutans*, and *S. enterica*	Biofilm elimination by 60%	El-Deeb et al. [140]
PLGA	151 nm	Biofilm under the flow condition	*E. coli*	A 20-fold reduction of bacterial colony	Zhang et al. [141]
PLGA	240 nm	Biofilm-infected skin wound in diabetic mice	MRSA	Elimination of biomass by 67%	Hasan et al. [143]
Hyaluronic acid	174−194 nm	*P. aeruginosa* abscess model in mice	*P. aeruginosa*	A 4-fold reduction of bacterial colony in abscess	Kłodzińska et al. [145]
PLGA and chitosan	230 nm	MRSA-infected full-thickness wound in mice	MRSA	A 80% reduction of bacterial colony in skin wound	Wu et al. [146]
PLGA, PCL, and chitosan	217−263 nm	Ex vivo model of biofilm on pig skin	*P. aeruginosa* and *S. aureus*	More than 99% of bacteria is killed	Permana et al. [149]
PCL	199 nm	Ex vivo model of biofilm on pig skin	*P. aeruginosa*, *S. aureus* and MRSA	A 88−100% killing of bacterial aamount	Mir et al. [150]
Alginate	179 nm	Ex vivo model of biofilm on pig skin	*P. aeruginosa*	A reduction of bacterial viability in biofilm	Singh et al. [152]

MRSA, methicillin-resistant *Staphylococcus aureus*; PCL, poly(ε-caprolactone); PLGA, poly(lactic-*co*-glycolic) acid.

**Table 4:** The application of lipid-based nanoparticles for antibiofilm treatment.

Nanoparticle Type	Average Size	Infection Model	The Microorganisms Tested	Antibiofilm Efficacy	Reference
Liposomes	111 nm	In vitro biofilm susceptibility test	*S. aureus* and *Streptococcus epidermidis*	Biofilm growth inhibition	Eroğlu et al. [155]
Liposomes	55 nm	Subcutaneous infection in mouse skin	*S. aureus*	A 8-log reduction of bacterial colony in biofilm	Li et al. [156]
Nanoemulsions	Not determined	Burn wound in mouse skin	MRSA	A 84% killing of bacterial number in biofilm	Song et al. [158]
Nanoemulsions	78 and 85 nm	In vitro biofilm disk assay	*C. albicans*	Biofilm elimination by 80%	Lewińska et al. [159]
Liposomes and nanoemulsions	75 and 214 nm	Subcutaneous infection in mouse skin	*S. aureus*, *S. epidermidis*, and MRSA	A 2.4-fold reduction of biofilm thickness	Lin et al. [160]
NLCs	177 nm	Subcutaneous infection in mouse skin	MRSA	A 4-log reduction of bacterial colony in abscess	Alalaiwe et al. [161]
SLNs	About 300 nm	Burn wound healing study in rats	*P. aeruginosa*	Removal of 79% of biomass	Patel et al. [162]
Lipid-polymer nanohybrids	14 nm	Intradermal MRSA infection on mice	MRSA	A significant biofilm elimination determined by live/dead staining	Hassan et al. [163]
